# A qualitative study of experiences with physical activity among people receiving opioid agonist therapy

**DOI:** 10.1186/s13011-024-00607-9

**Published:** 2024-05-06

**Authors:** Einar Furulund, Siv-Elin Leirvåg Carlsen, Karl Trygve Druckrey-Fiskaaen, Tesfaye Madebo, Lars T Fadnes, Torgeir Gilje Lid

**Affiliations:** 1 Centre for Alcohol and Drug Research, Stavanger University Hospital, Stavanger, Norway; 2 Department of Addiction Medicine, Bergen Addiction Research, Haukeland University Hospital, Bergen, Norway; 3 Department of Global Public Health and Primary Care, University of Bergen, Bergen, Norway; 4 Department of Respiratory Medicine, Stavanger University Hospital, Stavanger, Norway; 5 Department of Clinical Science, University of Bergen, Bergen, Norway

**Keywords:** Substance-related disorder, Exercise therapy, Outpatient, Opioid-related disorder, Health services, Motivation

## Abstract

**Background:**

Physical or mental health comorbidities are common among people with substance use disorders undergoing opioid agonist therapy. As both a preventive and treatment strategy, exercise offers various health benefits for several conditions. Exercise interventions to people with substance use disorders receiving opioid agonist therapy are limited. This study aims to explore experiences with physical activity, perceived barriers, and facilitators among people receiving opioid agonist therapy.

**Method:**

Fourteen qualitative interviews were conducted with individuals receiving opioid agonist therapy in outpatient clinics in Western Norway.

**Results:**

Most were males in the age range 30 to 60 years. Participants had diverse and long-term substance use histories, and most received buprenorphine-based opioid agonist therapy. The identified themes were (1) Physical limitations: Participants experienced health-related problems like breathing difficulties, pain, and reduced physical function. (2) Social dynamics: Social support was essential for participating in physical activities and many argued for group exercises, but some were concerned about the possibility of meeting persons influenced by substances in a group setting, fearing temptations to use substances. (3) Shift in focus: As participants felt the weight of the health burden, their preference for activities shifted from sports aiming for “adrenaline” to a health promoting focus. (4) COVID-19’s impact on exercise: because of the pandemic, group activities were suspended, and participants described it as challenging to resume. (5) Implementation preferences in clinics: Not interfering with opioid medication routines was reported to be essential.

**Conclusion:**

This study offers valuable insights for the development of customized exercise interventions aimed at enhancing the health and well-being of patients undergoing opioid agonist therapy. These findings underscore the significance of addressing social dynamics, overcoming physical limitations, and implementing a practical and effective exercise regimen.

**Supplementary Information:**

The online version contains supplementary material available at 10.1186/s13011-024-00607-9.

## Background

More than half of all patients with substance use disorders (SUD) receiving opioid agonist therapy (OAT) have somatic or mental health comorbidities [1]. In a recent Norwegian cohort study of people receiving OAT, half of the population reported at least seven somatic complaints, and the reported somatic burden was associated with a higher level of mental distress [2]. Non-communicable diseases (NCDs) such as cardiovascular diseases, chronic obstructive pulmonary disease (COPD) and cancer, appear earlier than in the general population [3, 4]. Considering OAT reduces mortality and overdose risk [5], the clinical focus needs to shift and recognize that NCDs reduces quality of life and life expectancy.

The benefits of physical activity and exercise on the general population’s somatic and mental health are well documented [6, 7]. Physical exercise, which is generally considered safe, is not only used as a preventive strategy to reduce health challenges or illness, but also as adjunctive therapy for several conditions including COPD, cancers, and schizophrenia [8–10]. Engaging in physical exercise offers a effective strategy for individuals suffering from SUD, with the ability to enhance rates of abstinence, mitigate withdrawal symptoms and reduce symptoms of anxiety and depression [11, 12]. Nevertheless, exercise-related interventions within this population have proven to be challenging with low adherence rates and some individuals having negative experiences to exercise [13–15]. Further, there is limited research on how to best implement exercise interventions among people receiving OAT [16–18]. A recently published systematic review identified only six intervention studies on physical activity and exercise among people in OAT [16], most of which had methodological shortcomings and were not sufficiently sized to evaluate the effects [19–24]. Several of these studies also reported difficulties in adherence, indicating that the interventions were not sufficiently adapted to the participants and did not take into consideration the group’s barriers and facilitators.

Therefore, this study aims to explore patients’ experiences with physical activity and exercise in opioid agonist therapy. The study further aims to improve the understanding of barriers and facilitators of physical exercise for patients in opioid agonist therapy clinics.

## Materials and methods

### Design

This is a qualitative study using semi-structured interviews. The study was designed as part of the ATLAS4LAR project [25], which has established a cohort and a health registry for OAT patients in Stavanger and Bergen. The interview guide, collaboratively created with clinicians and people with user experience, also encompasses topics related to participants perspectives on nutrition and smoking [26]. This paper presents the findings related to physical activity. A COREQ checklist was applied to ensure quality and transparency in the reporting, see supplementary file [27].

### Participants and setting

The interviews were conducted in two of the largest cities in Western Norway with a combined population of approximately 400,000 people. In both cities, there are several OAT outpatient clinics with a multidisciplinary team of health and social workers. The clinical staff monitored the intake of OAT medications such as buprenorphine or methadone weekly. During the COVID-19 pandemic, the Norwegian government and health authorities implemented closures of schools, non-essential businesses, and various public facilities during lockdown. Furthermore, individuals were advised to minimize face-to-face interactions and social gatherings, work remotely, limit travel, and avoid crowded places [28]. These measures were continuously assessed and adjusted according to the situation and circumstances of the ongoing infection. As a result of the pandemic, adjustments were made to clinical practices, which included extending the intervals for take-home medications, decreasing the frequency of supervised dosing, and enhancing remote support through expanded use of telephone and video consultations [29]. As a purposive sample, we included OAT clients in Bergen and Stavanger who reflected the clinics’ age and gender distributions. We aimed to recruit patients both with and without comorbid SUD and with various levels of motivation to change health habits. With weekly follow-up from their OAT clinic and included both active and less active individuals. Each year, research nurses at the OAT clinics offer health assessments to patients. Following the annual health assessment, participants’ thoughts on lifestyle and lifestyle changes were explored in an individual qualitative interview. There were no exclusion criteria other than not being able to take part in an interview in Norwegian. For more information about the participants, see Table 1.

### Data collection

Three female research nurses, trained in qualitative interview, performed all fourteen interviews in the clinics during January and February 2021. They were instructed to let the patients choose the sequence of topics and move between topics and questions as the interview progressed. Participants were informed that the interview would cover three topics: smoking, nutrition, and physical activity. The interview guide was not modified during the data collection. The interviews lasted approximately 40 to 60 min and were audiotaped and transcribed verbatim by the first three authors. We initially invited approximately 20–25 patients for interviews. 18 agreed to participate, and 14 attended the scheduled sessions. Among those who attended, 13 completed the interview, while one participant withdrew after 14 min.

Due to the ongoing COVID-19 pandemic, the interview setting was designed to minimise the risk of virus transmission. This included maintaining distance, and sometimes using facemasks. Before patients entered the OAT clinic for interviews, they were screened for Covid symptom by using a symptom checklist to assess their health status. This was conducted by healthcare personnel.

The patients were recruited by phone or after an appointment at the clinic, and sometimes the research nurses collaborated with clinical staff in contacting patients. All participants had met the research nurses in the annual health assessment at least once before the interview, where they received information about the study, and some were recruited during these sessions.

### Data analysis

The first three authors transcribed verbatim each recording with a pseudonym reflecting the gender of the participants. We used systematic text condensation in the analytical process [30], with the following four steps. Firstly, the authors read the transcripts to gain an overview and identify preliminary themes. Secondly, code groups were developed in collaboration in a workshop. Thirdly, subgroups exemplifying aspects of each code group were identified and condensed, and relevant quotes were identified. Finally, the condensates were synthesised to generalise descriptions and concepts of patients’ experiences with physical activity. NVivo 12 were used in the analysis process, and Microsoft Teams were used to facilitate digital collaboration during the coding process. The first author led this process, with close collaboration and supervision from co-authors.

## Results

All participants had injected drugs at some time in life, and most received buprenorphine-based OAT. Most were males in the age range of 30–60 years, with a median age of 49. All participants lived in stable housing conditions, and the majority lived together with others (Table 1).

Themes that emerged during the analysis were: (1) Physical limitations, (2) COVID-19’s impact on exercise, (3) Social dynamics, (4) Shift from adrenaline-driven activities to a health promoting focus, (5) and preferences for exercise interventions in OAT.

**Table 1:** Characteristics of the participants

	*n*
Female/male	3 / 11
OAT medication,	
* Methadone*	4 of 14
* Buprenorphine*	10 of 14
Education	
* Not completed basic education* ^*1*^	3 of 14
* Completed basic education* ^*1*^	5 of 14
* High school* ^*2*^	4 of 14
* University*	2 of 14
Stable housing conditions	14 of 14
Living alone	6 of 14
Debut age, median (range)	
* Alcohol*	13 (10–17)
* Cannabis*	14 (12–30)
* Stimulants*	23 (15–32)
* Opioids*	25 (14–32)
* Benzodiazepines*	19 (13–45)
* Tobacco*	13 (10–27)
Injected in the past 6 months	5 of 14

^1^ In Norway, the first 10 years of school are mandatory. ^2^ Grades 11–13

### Hurdles of health

Several participants talked about feeling vulnerable and fragile as adults because of their health situation. They described how current health problems, such as having a knee pain, back pain, or reduced lung function, prevented them from being active or exercising. In addition, several participants mentioned that shortness of breath was a problem, and some avoided walking uphill due to these challenges. Participants also explained that they struggled with coordination and concentration when being physically active; sometimes, they were afraid of falling and/or being injured during the activity.

*“I have no breath … whenever I try to do something [physical], my lungs stop working, and I can’t get enough air.” (*Oliver).

### The double-edged sword of social dynamics

According to the participants, being social and meeting people are essential for staying active. Some described close relationships as necessary to participate in activities. The support of partners or cohabitants was also essential regarding increasing physical activity and maintaining a healthy lifestyle.

*“He [the clinician at the OAT clinic] was brilliant. He was very into it [physical activity], and he knew what I was going through. We went for a walk once a week, him and me. After that, we drank coffee, chatted, and enjoyed ourselves.”* (Erik).

Participants talked about the possibility of going to their local gym, which for several were perceived challenging. They were concerned about not knowing how to use the equipment or feeling inadequate on their own. Several also discussed group dynamics and how they needed to feel safe and secure when interacting with their peers. Additionally, some expressed a desire not to exercise with those who were under the influence of substances.

*“My friend joined the group [a user organization offering physical activity], and then suddenly someone came with a hanging head [being sedated by substances], and I thought, no, I cannot join things like that. Then, it’s wasted.”* (Jacob).

### Shift from adrenaline-driven activities to a preventive focus

When growing up, many had experienced physical exercise or sports such as swimming, soccer, cycling and hiking. Many were familiar with exercise or physical activity through treatment facilities as adults. Throughout their lives, several commented that their view of exercise had changed. In previous years, exercise preferences shifted from high-adrenaline activities to a more preventive focus.

*“Being physically active makes me very happy. As a result, I have mostly had positive experiences with exercise and increasing my endurance.*” (Kristian).

Others felt they needed to invest in their health as they aged. Being healthy was important to them, as they hoped to live long. Some participants said they already focused on improving their health by increasing their daily activity and changing their daily routines.

*“I truly do a lot to improve my everyday health. I rarely take the bus, I walk a lot, and I am restless, so I like to go for a lot of walks. I also exercise from time to time, so I am investing in my health.”* (Thomas).

In addition, some explicitly mentioned that they wanted to improve their lung capacity, while others said there was a need for better routines, strategies, and self-discipline to make progress in their exercise.

“*I know I‘m not twenty years old, so I should invest in my health before I get old. It [exercise] can lift you mentally, and it can lift you physically. There are, in a way, only gains in it if you have the motivation to continue with it [exercise]*.” (Thomas).

### COVID-19’S impact on exercise

Many participants wanted to resume exercising when the COVID-19 lockdown and restrictions were lifted. Nevertheless, after two years of pandemic measures, participants pointed out barriers and opportunities for training. Some had the opportunity to train at home; however, they lacked the self-discipline and knowledge to exercise effectively and needed an instructor to lead the sessions. For others, the economy was a barrier especially if they wanted to train at a gym, as a membership fee was hard to prioritise within a tight budget. Additionally, some highlighted the joy of low-cost activities such as walking with family or friends.

*“Although I could do yoga at home, I prefer to have someone guiding me. If I do not, I get a little insecure and everything gets unorganized.”* (Jens).

Group exercises were resumed and closed repeatedly to adapt to the changing COVID-19 restrictions. The lockdown led to several participants not returning to their weekly exercise routines after reopening. Several felt it was meaningful to be part of an exercise group and were concerned about their health when they reduced their participation in weekly activities and routines. Feeling guilty about not exercising and letting the group down became for almost a habit, which affected their mental health negatively.

*“…there have been periods since the COVID-19 pandemic started when I have not trained at all. Additionally, I dropped out of the daily rhythm… It becomes almost a habit, and you begin to feel guilty if you do not work out regularly.”* (Thomas).

### Specific preferences for exercise interventions in the clinics

Participants were explicitly asked about their preferences for establishing training facilities at the OAT clinic. Due to the delay from medication intake until experiencing an effect, the majority expressed a reluctance to exercise too early in the morning. The group consensus was to start the exercise session around the middle of the day. Many participants missed having a fixed appointment on their weekly schedule. Therefore, an essential element would be establishing routines with fixed training days. All participants wanted to exercise with mixed genders, and several male participants said they interacted more easily with females than men. One female participant stated that activity with both sexes was acceptable; nevertheless, being the only female was not considered ideal. Regarding supervisors, participants did not prefer specific professions to supervise the sessions. Interestingly, few participants suggested user involvement or peer-led exercise groups within the clinical context. About the content of activities, little attention was given to the element of joy or having fun when exercising. However, they expressed the need for variation in content within the session. This would ensure that their performance would be based on their abilities and positively affect their health.

## Discussion

Results showed that participants perceived physical limitations as important barriers to starting exercise. However, having physical limitations could also improve motivation and to start exercising in order to reduce these. With declining health and turning older, participants’ activity preferences also shifted toward health prevention. Exercise as a social arena with both positive and negative aspects were highlighted. Moreover, they found that the pandemic had created a new barrier: after group activities were suspended, they had difficulty participating once the activities resumed.

Physical activity is widely used as adjunctive therapy for people with SUD, both therapeutically and to improve health [10, 31]. However, there are still gaps in the literature concerning how to best implement physical activity in OAT clinics, particularly in terms of adapting interventions and ensuring good adherence [16]. From our findings, most participants had experienced a decline in health and daily function. They viewed exercise as a tool to improve their health. One concern is that their health status and functional abilities are barriers to becoming more physically active. Similarly, physical ailments seem to be the most important barrier among the older adults population, whereas health concerns are the strongest motivator to exercise [32]. Participants from our study expressed a need to invest in their health. Several explained how they struggled with reduced lung capacity and needed to adjust their lives accordingly. Wanting to be healthy and live a normal life were motivational factors participants explicitly mentioned. From these statements, one could argue that exercising facilitates a normal life by providing meaningful activity and social interaction for people in OAT. In that case, a higher-intensity training program with a combination of endurance and strength training is recommended to improve health, feel progress, and provide meaningful activity [7]. This combination aligns with previously reported preferences for people with opioid dependence [33]. In our study, participants wanted their training to impact their health by seeking not only an enjoyable social activity, but also an activity that would help them improve their mental and physical health.

Although participants had positive experiences with physical activity and a desire to use physical activity to increase their health and daily function, this was insufficient to start exercising. These findings are consistent with a recently conducted cross-sectional study of attitudes and preferences for people with opioid use disorder [33]. In that study, approximately 70% of patients wanted a group activity or had no specific preferences for group or individual training [33]. In our study, several mentioned they needed a social arena in their lives, and exercise may facilitate an opportunity for social interaction. Some may also need a sense of belonging and social support to overcome the threshold to start exercising. Participants’ physical vulnerabilities, including pain and reduced lung function, increased their fear of injury during exercise, while after exercising, social challenges the gym and disruption to daily life due to pandemic restrictions contributed to lower participation and subsequent feelings of guilt. Before implementing physical activity, it may be necessary to discuss the group dynamics, rules, and functional abilities, such as coping with substance use. Group exercises offer two-sided possibilities: It could provide participants with social opportunities but also create negative experiences for them. In the current study, there may be a risk that a group member is under the influence of substances, potentially making other group participants feel more insecure and more vulnerable to a relapse. This adverse social interaction may be one reason why almost one-third of people with opioid use disorder want to exercise alone [33]. According to our findings, group exercises have more positive than negative aspects. Social interaction could also increase social support and help participants to achieve better daily routines.

During COVID-19, most participants experienced a sudden transition in their lives, from some participated in group activities or exercised in a gym to feeling isolated and attempting to stay active with a home-based exercise. They mentioned a lack of supervision during the home sessions induced feeling of insecurity and disorganisation, reducing the workouts’ effectiveness and enjoyment. Several emphasised the value of being part of a group exercise. Furthermore, some felt guilt being absent from group activities, letting the group down and not being present. This suggests an underlying appreciation for mutual support provided in the group context. This finding underlines the inherent benefits of group activities when promoting physical well-being.

Participants expressed several specific preferences and considerations regarding the implementation of exercise interventions in OAT settings. Their preferred time to exercise was late in the morning or around noon after receiving their OAT medications. It was important that the physical activity did not negatively interfere with their OAT medication routines. However, an integrated treatment model should consider more than just the patients’ perspective. Our findings highlight that participants prefer midday exercise sessions, aligning with their medication schedules and the time it takes to feel its effects. Furthermore, conducting these sessions at midday is practical when it is within the clinic’s operational hours. It is essential to understand the routines and resources at the clinic and their barriers, including the attitudes and knowledge of the clinical staff [31, 34]. The success of Integration of an exercise program is also influenced by factors such as organisational culture, leadership commitment, staff interest, and high service user demand [35, 36].

This study has several strengths and limitations. Participants were selected with purposive sampling based on a set of criteria. To recruit, one had to assume that the participant could participate in the interview and be able to reflect in the conversation. Additionally, we recruited participants receiving weekly follow-up from their OAT clinics and included both active and less active individuals. By establishing these criteria, one could make the case that we have omitted individuals with very high or very low physical abilities. This implies that our findings are likely applicable to the majority of individuals undergoing OAT, but may not fully extend to those at the extremes of functioning. A strength is that the sample is comparable in terms of age and sex to the Norwegian OAT population [37]. In addition to health professionals, people with user experience helped design the interview guide, which contributed to making it more relevant, understandable, and feasible for participants, adding to the study strength. On the other hand, there is a possibility that explicitly searching for information on health habits, other relevant information may be missed. Furthermore, social desirability biased may have added to this [38]. Participants may have been more open to social acceptance during the interviews because they were still actively engaged in rehabilitation clinics when data were collected. We also acknowledge the possibility of recall bias and its impact on the participants’ answers. However, we were mostly interested in regular behaviours and did not ask for one specific event or one type of specific exercise probably limiting recall difficulties.

## Conclusions

Our findings emphasize the importance of addressing social dynamics, overcoming physical limitations, and establishing a pragmatic and efficient exercise regimen. It is probable that these interventions will require pilot testing to refine their effectiveness and bridge the gap in the need for a sufficiently large intervention study to assess their impact.

### Electronic supplementary material

Below is the link to the electronic supplementary material.

Supplementary Material 1

## Data Availability

No datasets were generated or analysed during the current study.

## Abbreviations

SUD: Substance use disorder
OAT: Opioid Agonist Therapy
NCDs: Non-Communicable Disease
COPD: Chronic Obstructive Pulmonary Disease
