# Supplementary material for: A qualitative study of experiences with physical activity among people receiving opioid agonist therapy
Source: Subst Abuse Treat Prev Policy. 2024 May 6;19:26. doi: 10.1186/s13011-024-00607-9 (PMC11075283; doi:10.1186/s13011-024-00607-9)
Supplement: Supplementary file 1 — Supplementary Material 1 [file 13011_2024_607_MOESM1_ESM.docx]

**Consolidated criteria for reporting qualitative research (COREQ)(1):**

| Topic | Item No. | Guide questions/ descriptions | Responses |
| --- | --- | --- | --- |
| **Domain 1: Research team and reflexivity** | | |  |
| Interviewer/facilitator | 1 | Which author/s conducted the interview or focus group? | None. Interviews were conducted by research nurses affiliated with the research group |
| Credentials | 2 | What were the researcher’s credentials? *E.g. PhD, MD* | EF: M.Sc. and Phd-candidate, KLTD: MD and Phd-candidate,TM: MD Phd, SELC: M.sc. PhD, LTF: MD PhD, TGL: MD PhD |
| Occupation | 3 | What was their occupation at the time of the study? | All researcher were employed by the Bergen addiction research group in Helse Bergen or centre for Alcohol and drug research in Helse Stavanger. |
| Gender | 4 | Was the researcher male or female? | Both |
| Experience and training | 5 | What experience or training did the researcher have? | The senior researchers (SELC, LTF AND TGL) have previous qualitative research experience |
| Relationship established | 6 | Was a relationship established prior to study commencement? | All participants knew the research nurses from yearly health assessments |
| Participant knowledge of the interviewer | 7 | What did the participants know about the researcher*? e.g. personal goals, reasons for doing the research* | A brief explanation of the project (ATLAS4LAR) was presented to the participants. |
| Interviewer characteristics | 8 | What characteristics were reported about the interviewer/facilitator? *e.g. Bias, assumptions, reasons and interests in the research topic* | Female research nurses with qualitative interview experience. |
| **Domain 2: study design** | | |  |
| Methodological orientation and Theory | 9 | What methodological orientation was stated to underpin the study? *e.g. grounded theory, discourse analysis, ethnography, phenomenology, content analysis* | The study was analyzed using systematic text condensation. |
| Sampling | 10 | How were participants selected? *e.g. purposive, convenience, consecutive, snowball* | The sampling was purposive. |
| Method of approach | 11 | How were participants approached? *e.g. face-to-face, telephone, mail, email* | Participants were approached by telephone or face to face. |
| Sample size | 12 | How many participants were in the study? | 14 |
| Non-participation | 13 | How many people refused to participate or dropped out? Reasons? | There was no specific record of this |
| Setting of data collection | 14 | Where was the data collected? *e.g. home, clinic, workplace* | At the their local OAT- clinic |
| Presence of non-participants | 15 | Was anyone else present besides the participants and researchers? | No |
| Description of sample | 16 | What are the important characteristics of the sample? *e.g. demographic data, date* | See Table 1: Characteristics of the participants |
| Interview guide | 17 | Were questions, prompts, guides provided by the authors? Was it pilot tested? | Open-ended questions were part of the semi-structured interview guide. The guide was not piloted, but created in collaboration with researchers, health care professionals and user representatives. |
| Repeat interviews | 18 | Were repeat interviews carried out? If yes, how many? | None were repeated |
| Audio/visual recording | 19 | Did the research use audio or visual recording to collect the data? | Interviews were audio-recorded |
| Field notes | 20 | Were field notes made during and/or after the interview or focus group? | Yes, some. |
| Duration | 21 | What was the duration of the interviews or focus group? | The interval was between 13-60 minutes. |
| Data saturation | 22 | Was data saturation discussed? | During the analysis process, the authors discussed saturation. |
| Transcripts returned | 23 | Were transcripts returned to participants for comment and/or correction? | No |
| **Domain 3: analysis and findings** | | |  |
| Number of data coders | 24 | How many data coders coded the data? | All authors coded preliminary themes. Once the themes were decided upon EF and KTDF extracted meaning units. |
| Description of the coding tree | 25 | Did authors provide a description of the coding tree? | As part of the supplementary |
| Derivation of themes | 26 | Were themes identified in advance or derived from the data? | Based on data, themes were identified. |
| Software | 27 | What software, if applicable, was used to manage the data? | The NVivio software was used to manage the data and Microsoft teams to collaborate. |
| Participant checking | 28 | Did participants provide feedback on the findings? | No, but user representative did. |
| Quotations presented | 29 | Were participant quotations presented to illustrate the themes / findings? Was each quotation identified? *e.g. participant number* | Quotes were identified by pseudonyms given to participants. |
| Data and findings consistent | 30 | Was there consistency between the data presented and the findings? | There is constancy between presented data and the findings. |
| Clarity of major themes | 31 | Were major themes clearly presented in the findings? | The major themes are clearly shown with each subheading |
| Clarity of minor themes | 32 | Is there a description of diverse cases or discussion of minor themes? | It discusses a few minor themes and cases. |

Developed from:

Tong A, Sainsbury P, Craig J. Consolidated criteria for reporting qualitative research (COREQ): a 32-item checklist for interviews and focus groups. Int J Qual Health Care. 2007;19(6):349-57.
